# The mechanisms and cross-protection of trained innate immunity

**DOI:** 10.1186/s12985-022-01937-5

**Published:** 2022-12-08

**Authors:** Shiwei Hu, Danhong Xiang, Xinlu Zhang, Lan Zhang, Shengjie Wang, Keyi Jin, Liangshun You, Jian Huang

**Affiliations:** 1 Department of Hematology, The Fourth Affiliated Hospital, Zhejiang University School of Medicine, Zhejiang University, Yiwu, Zhejiang China; 2 Department of Hematology, The First Affiliated Hospital, Zhejiang University School of Medicine, Zhejiang University, Hangzhou, Zhejiang China; 3 Zhejiang Provincial Clinical Research Center for Hematological Disorders, Hangzhou, Zhejiang China

**Keywords:** Trained innate immunity, Mechanism, Cross-protection, Infection, Cancer, Immune response

## Abstract

In recent years, the traditional cognition of immunological memory being specific to adaptive immunity has been challenged. Innate immunity can mount enhanced responsiveness upon secondary stimulation, and a phenomenon is termed trained innate immunity. Trained innate immunity is orchestrated by distinct metabolic and epigenetic reprogramming in both circulating myeloid cells and myeloid progenitor cells in bone marrow, leading to long-term resistance to related and non-related pathogens infections. The induction of trained innate immunity can also polarize innate immune cells towards a hyperresponsive phenotype in the tumor microenvironment to exert antitumor effects. This review will discuss the current understanding of innate immune memory and the mechanisms during the induction of innate immunity, including signaling pathways, metabolic changes, and epigenetic rewriting. We also provide an overview of cross-protection against infectious diseases and cancers based on trained innate immunity.

## Introduction

The vertebrate immune system has been classically divided into innate immunity and adaptive immunity. Innate immunity involves a set of cells, including monocytes, macrophages, neutrophils, dendritic cells (DC), and natural killer (NK) cells [1], which constitutes the evolutionarily first line of host defense and recognizes pathogen-associated molecular patterns (PAMPs) and damage-associated molecular patterns (DAMPs) through germline-encoded pattern recognition receptors (PRRs) in a rapid but nonspecific way [1, 2]. Adaptive immunity includes specialized B and T lymphocytes, characterized by lifelong immunological memory and specificity in recognizing antigens with high-affinity receptors [3, 4].

It has been convinced that immunological memory was an exclusive hallmark of adaptive immunity for a long time. Nevertheless, a growing body of literature has shifted this paradigm in the last decade, showing that the innate immune system can also exhibit memory characteristics [5]. In plants and invertebrate animals, which both lack adaptive immunity, protection against reinfection different from the target pathogen is observed in the first and subsequent generation [6–11]. An older study also demonstrated that an athymic nude mouse vaccinated with Bacillus Calmette–Guérin (BCG) resisted the secondary challenge of *Candida albicans*, a process mediated by macrophage activation [12]. Moreover, live attenuated vaccines can induce broad protection against heterologous pathogens in humans [13].

In conclusion, plants, invertebrates, and vertebrates can manifest increased responsiveness of innate immune cells upon nonspecific re-stimulation through persistent functional reprogramming, and a property termed trained innate immunity [14]. However, not all stimuli affect pro-inflammation. Lipopolysaccharide (LPS) can cause immune tolerance and weaken the intensity of the inflammatory response to re-stimulation, which is also an adaptation of host defense [15].

Compared with classical adaptive immunity, which depends on gene rearrangements of antigen receptors and production of lymphocyte clones, trained innate immunity exerts responsiveness by signaling pathways impinging on transcription factors and intricate interconnection between metabolic changes and epigenetic rewriting. Furthermore, it is interesting to note that although the memory of innate immunity is relatively short compared with adaptive immunity, studies have shown that trained innate immunity has a cross-generational effect, and the immune efficacy can last from 3 months to a year [16–18], which is at variance with the life span of mononuclear cells [19].

**Fig. 1:**
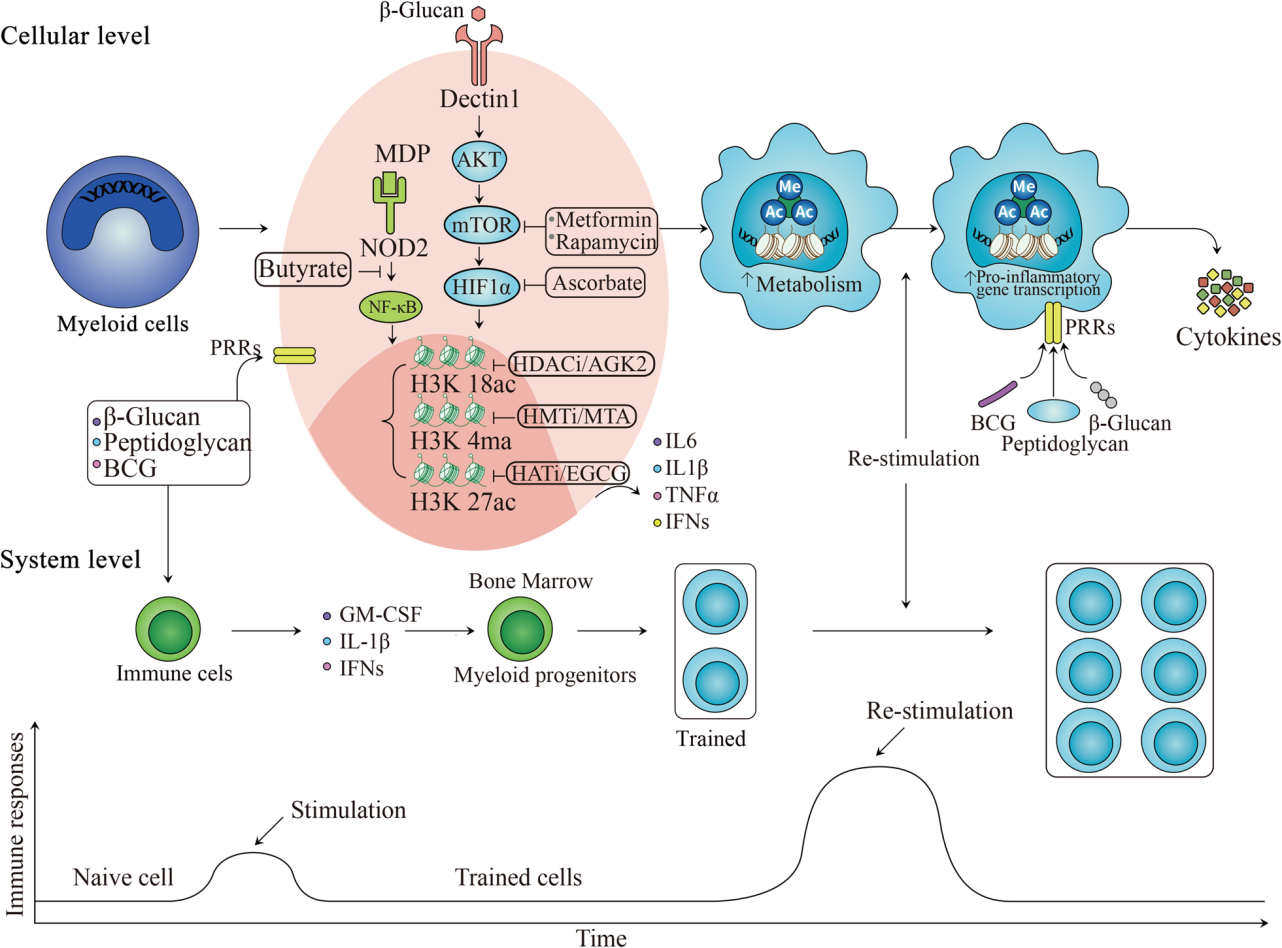
Mechanisms at the cellular and systemic level. Activation of the innate immune system can result in mild responsiveness, while it leads to enhanced responsiveness when challenged by subsequent heterologous triggers. This process is regulated at the cellular level (also known as peripheral trained immunity) and system-level (also known as centrally trained immunity). β-Glucan, MDP, and BCG are three of the most common inducers that contact the PRR of innate immune cells and release cytokines through signaling pathways, metabolic pathways, and epigenetic remodeling. β-Glucan recognizes the dectin-1 receptor and passes signal into cells via the AKT/mTOR/HIF1α pathway, promoting metabolic conversion from oxidative phosphorylation to aerobic glycolysis. Metabolites like fumarate can apply the inhibitory effect to the activity of KDM5, inducing the enrichment of H3K4me3 on promoters, and acetyl-CoA can also promote acetylation H3K27ac, resulting in an epigenetic rewriting. When stimulation is removed, some of the chromatin marks remain on histones and get ready for a faster modification and enhanced proinflammatory gene transcription upon re-stimulation, thus releasing more proinflammatory cytokines such as IL-6 IL-1β, TNFα, and IFNs. Rapamycin and metformin can nullify cytokine production via inhibiting mTOR, and ascorbate can abrogate trained innate immunity via inhibiting HIF1α. MDP and BCG can activate downstream NF-κB by interacting with intracellular NOD2 receptors, leading to epigenetic changes in H3K4me, H3K18ac, and H3K27ac. Butyrate can suppress the activation of trained innate immunity by preventing the acetylation of histone. Another important regulatory layer is HSPCs cells, which enable peripheral mature myeloid cells to be maintained for a longer period despite their short half-life. Inflammatory cytokines GM-CSF, IL-1β, and IFNs produced by innate immune cells can modulate myeloid progenitor cells in the bone marrow to promote myelopoiesis and generate trained monocytes. These two mechanisms play an important role in trained innate immunity

Defining the characteristics and mechanisms of trained innate immunity will better understand host defense and develop new avenues for preventing and treating diseases. This review will summarize the inner connection in the trained innate immunity regulation process (shown in Fig. 1) and provide a vision of its applications in infectious diseases and cancers.

## The mechanisms of trained innate immunity

### Mechanisms on a cellular level

#### Signaling pathway

Up to now, several signaling pathways associated with trained innate immunity have been discovered. Here, we will discuss two well-understood pathways led by the dectin-1 receptor and NOD2 (nucleotide-binding oligomerization domain-containing protein 2) receptor. Commonly, β-glucan-induced trained innate immunity is mediated by dectin-1/Akt/mTOR (mechanistic target of rapamycin)/HIF1α (hypoxia-inducible factor 1α) pathway, while MDP and BCG drive trained innate immunity through NOD/NF-κB (nuclear factor kappa-B) pathway [12, 20, 21].

Dectin-1 is a transmembrane receptor that can recognize β1,3-linked and β1,6-linked glucans via a lectin-like carbohydrate recognition domain [22]. β-Glucan is a polysaccharide component of fungal cell walls. When encounters with β-glucan, dectin-1 actives Akt, mTOR, and HIF1α successively to achieve the purpose of training monocytes. Intriguingly, the interaction between HIF1α and glycolysis leads to a forward loop, in which succinate activates HIF1α [23] and HIF1α promotes glycolysis by elevating pyruvate, increasing glucose consumption and lactic acid production in turn [20]. Using the inhibitors of the mTOR pathway, rapamycin or metformin, nullify the cytokine production [24, 25], and ascorbate (HIF1α inhibitor) can also abrogate trained innate immunity in a dose-dependent manner [20].

Researches have shown the emerging roles of NLRs in antiviral innate immune signaling pathways [26]. BCG and MDP (a common motif contained in all bacteria) can agonist NOD2, an intracellular sensor that belongs to the nod-like receptor (NLR) family [27]. NOD2 activation and signaling through nuclear factor kappa-B (NF-κB) stimulates epigenetic rewriting of macrophages like H3K4me, H3K18ac, and H3K27ac and induces trained innate immunity (showed in Fig. 1). NOD2-dependent activation of the NF-κB proinflammatory cascade of IFNs, TNF-α, IL-6, and IL-1β plays a role in resisting subsequent stimuli [12, 21]. When induced by BCG, there is no enhanced response in cells with NOD2 deficiency [12, 21]. The ubiquitination of RIP2 promotes the transcription of NF-κB. Thus, the inhibition of Rip2 kinase can impair training. Studies also demonstrated that Butyrate could suppress the activation of macrophages by preventing the acetylation of histones [12, 28].

#### Metabolic pathway

Cellular metabolism has emerged as a major biological node in maintaining cell homeostasis, proliferation, and cell-specific functions by providing energy and macromolecular building blocks [29]. Arts et al. assess network-level metabolome and transcriptome in primary human isolated monocytes induced by β-Glucan. The data identify several indispensable metabolic pathways contributing to trained innate immunity, such as glycolysis, glutaminolysis, and cholesterol synthesis [25]. When inhibiting one of these pathways, there is a significant decrease in the production of cytokines. In these pathways, many intermediate metabolites serve as a bridge between metabolic and epigenetic processes by acting as substrates or cofactors to regulate epigenetic enzyme activity [30]. Examples are abundant. Acetyl-CoA is the substrate for histone deacetylase. Fumarate and α-ketoglutarate are cofactors for histone lysine-specific demethylase 5 (KDM5) and JMJD3 (also known as KDM6), respectively [30]. The metabolic pathway is a critical mediator of epigenetic reprogramming dependent on trained innate immunity. It is well established that metabolic rewriting of innate immune cells will regulate the plasticity and epigenomic reprogramming in the context of trained innate immunity [31]. Here, we will briefly introduce major metabolites engaged in these processes.

The upregulation of glycolysis is mediated by dectin-1/Akt/mTOR/HIF1α pathway in monocytes trained with a high concentration of β-glucan [20], marked by a shift from oxidative phosphorylation to aerobic glycolysis [24]. Glycolysis provides pyruvate that goes into a tricarboxylic acid cycle (TCA) by converting to acetyl-CoA, an acetyl donor for histone acetyltransferases. Genes involved in glycolysis are epigenetically modified with activating histone marks. With the upregulating of acetyl-CoA in the context of trained innate immunity, acetylation of the genes of hexokinase and lactate dehydrogenase can promote glycolysis in turn [32]. However, 2-DG, an inhibitor acting on hexokinase, can block the training process of innate immunity, leading to suppressed IL-1β [33]. In β-glucan-activated monocytes, the accumulation of fumarate can apply the inhibitory effect to the activity of KDM5, inducing the enrichment of H3K4me3 on promoters, thus exerting influences on epigenetic reprogramming and increasing production of proinflammatory cytokines to subsequent stimulation [25]. Glutaminolysis results in the accumulation of α-ketoglutarate, which replenishes the TCA cycle. In LPS-activated macrophages, the production of α-ketoglutarate facilitates endotoxin tolerance through Jmjd3-dependent regulations and contributes to the M2-promoting mechanism [34]. In the same setting of macrophages, succinate is strongly upregulated to develop an enhanced proinflammatory response through HIF1α/IL-1β pathway [23]. The α-ketoglutarate/succinate ratio is the decisive factor in the transition from the M2 phenotype to the M1 phenotype, with a low ratio promoting inflammation [34].

Glycolysis can promote cholesterol synthesis, thus leading to the intracellular accumulation of an essential metabolite, mevalonate. It induces and amplifies trained innate immunity by increasing the expression of H3K4me3 on IL-6 and TNFα promoters through the IGF1R-Akt-mTOR pathway. Inhibition of the cholesterol synthesis pathway by 3-hydroxy-3-methyl-glutaryl coenzyme A reductase inhibitor (HMG-CoAi) like fluvastatin prevented the induction of training for β-glucan and BCG, indicating that the cholesterol synthesis pathway is essential for trained innate immunity [33]. Further evidence showed that patients with deleterious mutations in mevalonate kinase carried endogenous trained innate immunity phenotypes in monocytes, with increased upregulation of glycolysis, epigenetic changes, and inflammatory cytokines production [33].

Itaconate is another essential TCA cycle metabolite derived from cis-aconitate with IRG1 protein responsible for its generation, and its concentration is highly upregulated in LPS-activated macrophages [35]. An anti-inflammatory effector induces immune tolerance by inhibiting mitochondrial metabolism and cytokines such as IL-6 and IL-12 [36]. Three mechanisms account for the pronounced tolerizing effects. The major one is the inhibition of succinate dehydrogenase-mediated oxidation of succinate to fumarate, resulting in the elevated expression of KDM5 and decreasing of H3K4me3, which can lead to a state of immunoparalysis deleterious to the host as a result of increased susceptibility toward secondary infections [37]. Secondly, it supports the activity of the NRF2, an anti-inflammatory transcription factor, which limits further inflammatory gene expression of IL-1β and downregulates the IFN response. [38]. Thirdly, it selectively regulates secondary transcriptional responses to TLR stimulation by inhibition of LPS-mediated IκB induction via a key mediator, ATF3 [36]. However, β-glucan can offset this immune tolerance by inhibiting IRG1. The counterbalance induced by β-glucan enhances succinate dehydrogenase activity by downregulating the itaconate production, thus elevating the fumarate accumulation, leading to a series of subsequent activation of trained innate immunity [35]. Itaconate is a crucial regulatory node between tolerance and trained immunity and could be employed as a therapeutic tool in sepsis or cancers.

#### Epigenetic remodeling

Epigenetic remodeling means heritable alternation in gene function without any change in DNA sequence, leading to a shift in phenotype, which is just the way how trained innate immunity allows cells like monocytes and macrophages to respond more or less strongly upon secondary challenge. Current studies have shown that epigenetic reprogramming can occur in histone modification, non-coding RNA, and DNA methylation. Histone modification refers to an enzymatic modification such as methylation, acetylation, and phosphorylation. Several genetic markers are associated with trained innate immunity: H3K4me3, marking active promoters, H3K4me1, observed at the enhancers, and H3K27ac, characterized in both promoters and enhancers [20, 39]. Histone modifications can induce a proinflammatory phenotype in cells, but how do they play a role in trained innate immunity upon secondary stimulation? When the native myeloid cells are first stimulated, they are rapidly activated and upregulate gene transcription, associated with activating histone modification, characterized by a shift from high DNA methylation to low DNA methylation and an absence of histone modifications to an active expression of H3K4me3 and H3K27ac. Then, when stimuli are removed, these depositions of chromatin marks still partially rest in histones like H3K4me1, which is a notable feature since these quiescent myeloid cells can enhance gene expression and lead to a more robust cytokine production with re-stimulation. Based on this mechanism, it is worthy of attention to epigenetic enzymes (histone methyltransferase; histone demethylase; histone acetyltransferase; histone deacetylase) that can write or erase epigenetic markers. It is known that KDM5 and Lysine methyltransferase (Set7) play a key role in β-glucan-induced trained immunity. When the monocytes are exposed to β-glucan, the expression of KDM5 is downregulated by the accumulation of fumarate, followed by the persistence of H3K4me3 activity on the enhancers of specific metabolic enzymes [25]. While histone methyltransferase Set7 is enhanced, activating the deposition of histone modification H3K4me1 and opening the chromatin needed for the production of proinflammatory cytokines upon re-stimulation [40].

A recent study found that immune gene-priming lncRNAs, UMLILO underlies the molecular basis of trained innate immunity. In addition to the connection between β-glucan and the dectin-1/AKT/mTOR pathway, β-glucan can also activate NFAT signaling by triggering calcium influx via dectin-1, which permits NFAT to translocate into the nucleus. The increased level of NFAT on the UMLILO promoter results in an upregulation of its transcription. Finally, WDR5–MLL1 complex is recruited by UMLILO to certain promoters, such as IL-8, CXCL1, CXCL2, and CXCL3, to facilitate H3K4me3 expression on target genes [41]. As such, lncRNAs play a role as transporters for methyltransferases in the chromatin that contains trainable genes to link epigenetic and metabolic changes in trained innate immunity. Notwithstanding, how to modify the innate immune response genes by regulating UMLILO activity and how other stimuli other than β-glucan induce trained innate immunity through lncRNAs to warrant further studies.

As for DNA methylation, relevant research is scarce, and there needs to be an investigation into the function of DNA methylation in the development of trained innate immunity. A study among BCG-vaccinated subjects revealed that DNA methylation patterns on promoters of inflammatory genes were lost in a wide range of responders characterized by containment of *Mycobacterium tuberculosis* replication compared with non-responders [42]. Another study indicated that DNA methylation changes are restricted to a small scope of the genomic region compared with histone modification. And the majority of DMR appears at distal elements marked by H3K4me1. DNA methylation changes can be utilized as a biomarker for LPS-induced tolerance in macrophages [15], an exciting new field awaiting exploration.

### Mechanisms at a system level

In the last section, we have discussed the fundamental mechanisms underlying the enhanced response upon re-stimulation of myeloid cells. These trained myeloid cells can present protective functions for several months and even years [18]. However, it is acknowledged that mature myeloid cells are relatively short-lived in both mice and humans, with a lifespan of only a few days [43]. So here comes the puzzle: how can circulating myeloid cells persist much longer than their half-life period. Recently, more researchers have focused on the system level of training as a potential explanation for the puzzling question.

Trained innate immunity on a systematic level (also known as centrally trained immunity) modifies HSPCs in the bone marrow. Indeed, recent work showed that trained innate immunity could occur in HSPCs in addition to mature myeloid cells in the circulation. β-Glucan and BCG can reprogramme myeloid-biased HSPCs to generate trained innate immunity within the myeloid cell compartment. Mitroulis et al. administrated β-glucan to mice and observed increased expansion of hematopoietic progenitors and multipotent progenitors, which was essential for enhanced protection against subsequent challenges [44]. This phenomenon depended on inflammatory signaling, IL-1β and GM-CSF, and two major metabolic processes, glycolysis and cholesterol metabolism, similar to trained monocytes. β-Glucan-trained granulopoiesis can be activated and transmitted by bone marrow via Type I IFN signaling [45–47]. BCG-induced mouse model showed training and expansion of HSPCs and acquiring epigenetic and transcriptomic signatures such as H3K4me3 and H3K27ac. Moreover, myeloid-based HSPCs and bone marrow-derived macrophages could protect mice against subsequent *M. tuberculosis* infection [48]. Notably, naive mice were conferred with the ability to fight against reinfection after the bone marrow transplantation from BCG-trained mice, indicating long-term modulation of HSPCs. In addition to experiments in vitro, another clinical trial confirmed the same results that healthy volunteers vaccinated with BCG changed the transcriptional landscape of HSPCs. This program can be conveyed to circulating monocytes via epigenetic variation, which leads to a persistent trained immunity in peripheral cells [49].

Besides prototypical trained-immunity-inducing agonists like β-glucan and BCG, a recent study concerned whether sterile triggers like a Western-type diet can induce trained innate immunity. The research revealed that feeding a high-fat diet in Ldlr − / − (lack the low-density lipoprotein receptor) mice can produce prolonged transcriptomic and epigenomic reprogramming of mature myeloid cells and bone marrow myeloid stem cells via the NLRP3 inflammasome and IL-1β. Interestingly, systemic inflammatory markers were undetectable after 4 weeks of chow diet, while the trained immunity phenotype remained broadly augmented, confirming a long-lasting effect of trained innate immunity in the human body [50]. The role of reprogramming of HSPCs in other settings of trained innate immunity is under investigation.

## Trained innate immunity in infectious diseases

Trained innate immunity is characterized by providing cross-protection against infectious diseases following stimulation derived from bacteria, fungi, viruses, and parasites. This protective effect defends against a certain range of unrelated pathogens. Here, we will present preclinical and clinical trials aiming to explore this beneficial effect both in murine and humans (see Table 1).

**Table 1:** Trained immunity in infections

	Stimulant	Infections	Study type	References
Animal studies	*C. albicans*	*C. albicans*, *S. aureus*	Hybri (BALB/cCr x DBA/2Cr) mice	[51–53]
	BCG	*C. albicans*	SCID mice	[12, 54]
	BCG	Schistosoma mansoni	Nude mice	[55]
	BCG	Influenza A	Swiss Webster mice	[56]
	BCG	HSV	Swiss Webster mice	[57]
	MDP	Vaccinia virus, HSV	BALB/c and CDF1 (BALB/c x DBA/2) mice	[58]
	MDP	Sendai virus	BALB/c mice	[59]
	BCG	Japanese encephalitis virus	BALB/c mice	[60]
	BCG	Malaria	C57BL/6 mice	[61]
	β-Glucan	*S. aureus*	AKR/J mice	[62, 63]
	β-Glucan	Leishmania	C57BL6J and IL-32γTG mice	[64]
	MDP	*S. pneumoniae*, Toxoplasma gondii	Swiss Webster mice	[65]
	Herpes virus	Listeria monocytogenes, Yersinia pestis	C57BL/6 J mice	[66]
	Nippostrongylus brasiliensis	Corresponding reinfection	BALB/c mice	[67]
	IL-1	Pseudomonas aeruginosa	Swiss Webster mice	[68]
	CpG oligodeoxynucleotide	Meningitis (*E. coli*)	C57BL/6 mice	[69]
	Flagellin	*S. pneumonia*, rotavirus	BALB/c, C57BL/6 J, and outbred NMRI strain	[70, 71]
Human studies	BCG	Respiratory tract infections, neonatal sepsis, fever	Randomized controlled trial	[72, 73]
	BCG	Acute lower respiratory tract infection (RSV)	Case–control study	[74]
	BCG	Upper respiratory tract infection	Phase II, Randomized controlled trial	[75, 76]
	BCG	Pneumonia	Prospective study	[77]
	BCG	SARS-CoV-2	Clinical trial	[78–80]
	BCG	Yellow fever viremia	Randomized controlled trial	[81]
	BCG	Cutaneous and genital warts (HPV)	Randomized controlled trial	[82, 83]
	BCG	Influenza A and HSV	Randomized controlled trial	[84]
	BCG	Malaria	Randomized controlled trial	[85]
	BCG	*M. tuberculosis*, *C. albicans*, *S. aureus*, *E. col*	In vitro	[12, 18]
	HBV	Pseudomonas aeruginosa, Salmonella typhimurium, uropathogenic *E. coli*, Listeria monocytogenes, Acinetobacter baumanii	In vitro	[86]
	Chitin	*C. albicans*, *S. aureus*, *E. coli*	In vitro	[87]

### Implications of cross-protection in murine

Protection against infection could be mediated through education of the innate immune system to mount a more effective immune response against related and unrelated infections. Researchers chose experimental models that had a deficiency in adaptive immunity to validate the protective effect of innate immunity. In athymic and RAG1-deficient mice infected with an attenuated strain of *C. albicans* (PCA-2) conferred considerable protection against subsequent challenges with virulent strain CA-6 and even *Staphylococcus aureus*, demonstrating the protection is non-targeted and independent of lymphocytes [51–53]. Another study focused on SCID and NSG mice that both are absent of functional T cells and B cells, the latter lacking functional NK cells. Immunization of mice with BCG 2 weeks prior to inoculation of lethal *C. albican* dose, all the SCID mice lived through a 28-day observation period, while NSG mice only had a survival rate of 30%, confirming that BCG-induced protective effect of trained innate immunity was partially dependent on NK cells [12, 54]. The hypothesis that trained innate immunity can be elicited in adaptive immunodeficient mice was further supported by studies investigating the mechanism of protection against *Schistosoma mansoni* conferred by BCG vaccination in “nude” mice without mature T lymphocytes [55].

In addition to direct evidence, investigations in mice with normal immune function also demonstrated enhanced protection against secondary infections provided by trained innate immunity. The evidence indicated that BCG or MDP offered protection against secondary viral infections, such as influenza A virus, herpes simplex virus type 2 (HSV2), vaccinia virus, Sendai virus, and Japanese encephalitis virus. BCG can exert strong effects on the innate immune component of host defense. Spencer et al. showed that mice who received BCG had a lower viral titer of influenza A virus than placebo, mediated by macrophages and independent of IFN-γ [56]. These findings were in line with the results discovered by Starr et al. Administration of BCG 6 days prior to insults with HSV2 was found to reduce the risk of neonatal HSV infections and thus prolonged survival of newborn mice [57]. Intriguingly, BCG was able to relieve the neuropathological symptoms of the Japanese encephalitis virus and increased the survival rate of vaccinated mice, suggesting that BCG vaccination can induce protection in remote organs like the nervous system [60]. Receiving MDP subcutaneously or orally could enhance antiviral activity against vaccinia virus and HSV2 infections in mice, a process that was mediated by peritoneal macrophages [58]. Similarly, MDP also offered protection in the murine model of the Sendai virus with a possible role in macrophages [59].

Besides the function of cross-antiviral, cross-antibacterial is also a common effect of trained immunity that attributes to various ligands. Classic inducers like BCG, β-glucan, and MDP can protect against subsequent bacterial infection in a nonspecific manner. Peptidoglycan component MDP exerted protection against *Streptococcus pneumoniae* and *Toxoplasma gondii* [65]. BCG vaccination can increase protection against malaria due to intensified transcription of antibacterial proteins [61]. β-Glucan training led to protection against *S.* *aureus* sepsis and decreased S. aureus-associated lethality in normal and leukemic mice [62, 63]. β-Glucan also enhances Leishmania infection resistance via upregulated expression of IL-1 and IL-32 [64].

Researchers have discovered that other effective stimuli like viral and parasitic organisms and even proinflammatory cytokines may potentially induce trained innate immunity independent of the adaptive immune system. Herpes virus latency confers profound benefits to hosts' immune systems for its ability to resist Listeria monocytogenes and Yersinia pestis despite the risk of viral reactivation. This protection is achieved by long-term production of IFN-γ and activation of macrophages [66]. Parasitic nematode *Nippostrongylus brasiliensis* modifies macrophages to accelerate parasite clearance and reinfection [67]. Evidence also showed that prophylactic treatment with recombinant IL-1 in granulocytopenia mice led to protection against *Pseudomonas aeruginosa* [68]. Other examples show that flagellin can protect against systemic experimental infections like *S. pneumonia* and rotavirus [70, 71] and unmethylated CpG oligodeoxynucleotide contributes to preventing bacterial CNS infections such as meningitis caused by *Escherichia coli*, indicating the protection against reinfection can cross the blood–brain barrier [69]. In conclusion, these data indicated that the immune system’s exposure to microorganism-derived immune stimulatory agents could provoke protection against a subsequent challenge with the same or another pathogen. However, the results presented by some animal studies could partly be due to the priming effect instead of trained immunity since the secondary challenge in experiments was done within a week after primary stimulation, which warrants further validation and discussion.

### Implication of cross-protection in humans

Trained innate immunity’s cross-protection against infections has also been investigated in humans, and an increasing body of evidence suggests that BCG plays a key role here. Epidemiological studies reported that receiving BCG at birth can reduce mortality in neonates and improve the survival of low-weight infants. However, the main reasons are far beyond the reduction of tuberculosis mortality burden, which is supposed to lie in fewer cases of respiratory tract infections, neonatal sepsis, and fever, indicating the nonspecific protective effects of BCG in neonates [72, 73]. Additionally, a randomized controlled trial in healthy volunteers improved the positive effect of BCG against experimental human malaria infection [85], indicating a potential for malaria vaccine strategies. Finally, thirty healthy participants administrated with BCG 1 month before receiving the yellow fever vaccine resulted in decreased peak yellow fever viremia, which was correlated with an augmented proinflammatory activity of monocytes and mediated by 1L-1β [81]. All these data were crucial for designing vaccination programs and future vaccination strategies.

Notably, BCG showed a significant effect in protection against respiratory tract infection. Vaccination with BCG conferred an indispensable role of innate immune cells in host defense against acute lower or upper respiratory tract infection among people of all ages, including infants [74], adolescents [75], and even elder individuals [76]. Furthermore, a clinical trial showed that after administration of BCG in subjects with negative tuberculin test results, which meant depressed cell-mediated immunity, their previous high risk of pneumonia decreased to the same level as in tuberculin-positive older adults [77]. Based on the evidence that BCG could lead to respiratory viral and bacterial clearance, the hypothesis suggested a potential capacity of BCG vaccination to protect against SARS-CoV-2 infection by utilizing the trained innate immunity to reduce susceptibility [88]. Although epidemiological studies have suggested that countries with obligatory BCG vaccination had a lower mortality rate for COVID-19, evidence was still insufficient to support or deny this hypothesis due to some inherent biases [78–80]. Nowaways, more than 20 clinical trials in diverse populations are currently registered to inform on the effect of BCG vaccinations upon exposure to COVID-19. The ACTIVATE-2 study revealed BCG vaccine recipients had a lower risk to develop COVID-19 compared with placebo-vaccinated controls among patients older than 50 years with comorbidities [89]. While the latest phase 3 clinical trial conducted in South Africa founded that BCG revaccination failed to protect healthcare workers from SARS-CoV-2 infection or related severe COVID-19 disease and hospitalization [90]. Also, since there remained doubts in off-target effects of the BCG vaccine altered by subsequent administration of a different vaccin, WHO still recommendated that the BCG vaccine was used for COVID-19 only in randomized controlled trials [91].

In addition to the effect of protecting against respiratory infections, the protection by trained innate immunity against other viruses and bacteria is being extensively studied. Consistent with the outcomes in mice, BCG vaccination in adults could alter the clinical and immunological response to and herpes simplex virus [84]. Several trials indicated that BCG immunotherapy had a protective efficacy against cutaneous and genital warts caused by HPV [82, 83]. BCG vaccinated volunteers improved a positive effect against infection with *M. tuberculosis*, *C. albicans*, *S. aureus,* or *E. coli*, a process mediated by enhanced IFN-γ, TNF-α, IL-1β, and IL-6 produced by BCG-trained NK cells and peripheral blood mononuclear cells [18]. Besides, chitin-induced human monocytes exhibit an enhanced ability to protect against *C. albicans*, *S. aureus,* and *E. coli* [87].

Furthermore, it has been demonstrated that children born to HBV-infected mothers have a trained immunity profile featured by higher concentrations of IL-10, IL-6, IL-8, and TNF-α, which lead to an enhanced response to subsequent bacterial infections, such as *Pseudomonas aeruginosa*, *Salmonella typhimurium*, *uropathogenic E. coli*, *Listeria monocytogenes* and *Acinetobacter baumanii* [86]. The cross-protection against bacterial infections by trained innate immunity may represent a potential strategy for preventing surgical site infections since the most common cause was *S. aureus* [92]. It was speculated that trained innate immunity might prevent or reverse the postoperative immunoparalysis that contributes to the risk of infections following surgery [92].

## Trained innate immunity in cancer

### The role of trained innate immunity in TME

Efficient activation of the immune system is the foundation of eliminating cancer cells, but immune dysregulation can promote tumor progression and transformation. Innate immune cells infiltrating the TME can be reprogrammed to an anti-inflammatory phenotype, contributing to the immunoparalysis responsible for tumor progression [93]. Tumor-associated macrophages (TAMs) and MDSCs are two primary myeloid cells in the TME. They can adapt to various insults with substantial plasticity and have a dual role in promoting and inhibiting tumor growth, controlled by the signals emitted by cancer cells or stromal cells in the TME [94–97]. TAMs, which differentiate from monocytes infiltrating in the TME, facilitate tumor progression and suppress antitumor immune response through durable histone modifications [98–100]. MDSCs are a heterogeneous cell population derived from neutrophilic or monocyte precursors [101]. Epigenetic programs induced by long-term low-dose stimulation of chronic inflammation in the TME underlie a pro-tumorigenic profile in these cells [102, 103]. Trained innate immunity can overcome the immunosuppressive TME by rewriting the epigenetic programs of TAMs and MDSCs, indicating effective immunotherapy in cancer [104].

Considering the tumor tolerance exhibited by immunosuppressive myeloid cells, trained innate immunity-promoting therapies might prove clinically beneficial. As discussed above, trained innate immunity can rewrite the epigenetics of heterogenic myeloid cells to skew the M2 phenotype to the M1 phenotype with robust respiratory burst activity and phagocytosis, leading to proinflammatory and antitumor characteristics. Furthermore, it is beneficial to promote the number of “trained” monocytes to differentiate into antitumor macrophages, enhance the function and infiltration of T cells in the TME and increase the susceptibility to checkpoint-inhibitor drugs in oncotherapy [105].

Stimulants like BCG and β-glucan have long been immune adjuvants in treating many neoplasms. BCG vaccination is shown to have antitumor effects in the urothelial cell carcinomas and melanomas [106]. β-glucan also has evoked considerable interest in the immunotherapy of many solid and hematological malignancies [107]. The induction of β-glucan to MDSCs can contribute to the gain of enhanced antigen-presenting and loss of immunosuppressive phenotype by successfully reprogramming [108]. A previous study reported that repeated or persistent stimulation of LPS to macrophages epigenetically enforces tolerance. Nevertheless, whether vaccination with LPS can skew tumor-associated macrophages from M1 towards the M2 phenotype remains to be proven, but we foresee that LPS-induced tolerance of innate immune cells leads to immune paralysis and decreased transcription of inflammatory genes, placing the individual at greater risk of sepsis and tumor progression [109]. Here, we will discuss the evidence for trained innate immunity in cancer therapy-induced by BCG and β-glucan.

### BCG induces trained immunity in cancer immunotherapy

BCG vaccination plays an important role in preventing and treating cancer as an inducer of trained innate immunity. Infants vaccinated with BCG had a lower risk of developing melanoma and leukemia [110, 111]. BCG instillation after transurethral resection reduced tumor progression in non-muscle-invasive bladder cancer [112, 113]. The antitumor process of BCG is mediated by molecules produced in the TME, which act as PAMPs and DAMPs to train myeloid cells. Transcriptional reprogramming is still the central part of trained innate immunity in these cells, leading to an enhanced proinflammatory phenotype [114]. Repeated instillation triggers local infiltration of macrophages, DC, NK cells, and robust cytokine production, including IL-6, IL-18, GM-CSF, IFN-γ, and TNF-α, thus impairing the tolerogenic milieu developed by carcinogenic cells and enhancing antitumor immunity [115, 116]. Furthermore, trained innate immunity with BCG stimulus can lead to adaptive immune response, leading to the activation of T cells infiltrating the TME.

BCG has been extensively studied in the field of neoplasms as a practice of trained innate immunity in addition to bladder cancer, such as prostatic urothelial carcinoma [117], gastric cancer [118], colon cancer [119], head and neck squamous cell carcinoma [120]. Data from large epidemiological studies also reveal a potential for BCG in hematological malignancies. BCG vaccination during infancy could reduce the incidence of lymphoma, and a combination of BCG in the treatment of acute myeloid leukemia leads to a prolonged remission [121, 122]. In a guinea pig model of liver cancer, injecting the BCG vaccine improved the survival rate by 69% [123]. Another mouse-based experiment showed that the growth of MUC1-positive breast tumors was inhibited in subjects vaccinated with BCG [124]. A recent retrospective analysis of 2963 BCG vaccinees reported a statistically significant reduction in the risk of lung cancer development [125]. Further studies need to determine the dose and frequency of BCG vaccination, evaluate the degree of memory induced by trained innate immunity, and certify the capacity of innate immune cells to elicit adaptive immune responses.

### β-Glucan induces trained immunity in cancer immunotherapy

The antitumor characteristics of the fungal extract were first reported by Ringler [126], and since then, a large number of studies have been carried out to investigate the antitumor effect of β-glucan. Though researchers generally ascribe this therapeutic success to activated lymphocytes, it is quite likely that trained immunity mediated by metabolic and epigenetic reprogramming of innate immune cells contributes to the clinical response. The induction of β-glucan can convert the inhibitory M2 phenotype into a proinflammatory M1 phenotype and regulate the capacity of granulocytes and monocytes, resulting in cytokine secretion responsible for the recruitment and stimulation of other immune cells [100, 108, 127]. Interestingly, experiments showed an interconnection of autophagy and trained innate immunity induced by β-glucan [128]. Autophagy plays an indispensable role in the monocyte-macrophage differentiation, and the differential into M1 macrophages is extremely important in the antitumor activity [129]. So the complicated connection between autophagy, trained innate immunity, and antitumor activity warrants further investigation.

Β-glucan has been used as an immunostimulatory adjuvant in oncotherapy for a long time. Systemic treatment with β-glucan remarkably reduced the development of metastatic lung cancer, a process that was independent of canonically described β-glucan-dependent antitumor pathways and adaptive immunity [130]. In patients with myelodysplastic syndrome, the supplementation of β-glucan could enhance the function of neutrophils and monocyte [131]. Similarly, another study indicated that monocytes in patients with recurrent or metastatic breast cancer showed evident proliferation and activation after 2 weeks of oral β-glucan administration [132]. In cancer models of melanoma, the induction of β-glucan reduced the weight of the primary tumor significantly through the activation of NK cells [12].

Notably, researchers found that the combination of β-glucan with some conventional chemotherapeutic drugs promoted antitumor efficacy. BTH1677, a kind of β-glucan analog, improved objective remission rate with a combination of other chemotherapies in several neoplasms, including non-small-cell lung cancer [133], stage IV KRAS-mutant colorectal cancer [134], and acute lymphoid leukemia [135] when combined with other chemotherapeutic drugs. In a small phase I/II study, preliminary results showed that patients receiving additional treatment of β-glucan with primary chemotherapy had a beneficial effect on hematopoiesis. This combined therapy was well tolerated in patients [136]. Another trial conducted by Biothera et al. showed that conducting Imprime PGG (a β-glucan polymer) combined with cetuximab and irinotecan increased the response rate in advanced colorectal cancer [137]. In conclusion, the BCG vaccine and β-glucan in antitumor activity have been summarized in Table 2. Understanding the antitumor effect of β-glucan in the induction of trained innate immunity is still in its infancy, but we believe the applications of β-glucan alone or in combination with other immunotherapy will be an effective and promising strategy for cancer treatment in the future.

**Table 2:** Trained immunity in cancer

Stimulant	Cancer	Study type	References
BCG vaccine	Bladder cancer	Meta-Analysis	[138]
	Melanoma	Randomized controlled trial	[139]
	Lymphoma	Retrospective control study	[121]
	Acute myelogenous leukemia	Randomized controlled trial	[122]
	Breast cancer	SCID mice	[124]
	Liver cancer	Guinea pigs	[21]
	Prostatic cancer	Randomized controlled trial	[117]
	Gastric cancer	Randomized controlled trial	[118]
	Colon cancer	BALB/c mice	[119]
	Lung cancer	Randomized controlled trial	[140]
	Head and neck squamous cell Carcinoma	In vitro	[120]
Imiquimod	Basal cell carcinoma	Randomized controlled trial	[141]
Coley’s toxin	Sarcoma	Randomized controlled trial	[142]
Influenza virus	Lung cancer	BALB/c mice	[113]
β-Glucan	Lung cancer	C57BL/6 J mice	[130]
	Lymphoma	Randomized controlled trial	[136]
	Myelodysplastic syndrome	Randomized controlled trial	[131]
	Breast cancer	Randomized controlled trial	[132]
	Colorectal cancer	Randomized controlled trial	[137]
	Ovarian cancer	Randomized controlled trial	[143]
	Non-small-cell lung cancer	Randomized controlled trial	[133]
	Colorectal cancer	Phase II, Randomized controlled trial	[134]
	Acute lymphoid leukemia	BN/RijHsd rats	[135]
	Cervical cancer	Retrospective case–control study	[144]

## Discussion

In this review, the evidence presented above suggests that trained immunity endows the host with rapid and robust immune responses upon subsequent challenges, with inextricable and bidirectional links between metabolic and transcriptional pathways. Although there is an increasing body of arguments supporting the mechanisms at cellular and systematic levels, many unraveled questions remain to be addressed over the coming years. On the one hand, in-depth identification of the metabolic and epigenetic processes should be described. We need to have a deeper understanding of this immunological process in the entire range of cell populations and at a level of distinct stimulus and disease types. The diversity of stimuli and disease pathologies may influence the subsequent secondary phenotype. We believe effort in this field will benefit in figuring out the range of cross-protection and optimizing the therapeutic potential encompassed by trained innate immunity [145]. On the other hand, despite progress in the modulation of hematopoietic stem and progenitor cells, more precise details need to be explored in how chromatin landmarks can transmit steadily in chromatin disassembling and reassembling [17]. This warrants further investigation into the contributions of the germline in mammals, which influences the duration of innate immune memory [17].

With increasing lines of basic and translational studies, one of the most critical goals is to explore the effect of trained immunity on disease. We have demonstrated anti-infection and anti-cancer immune responses above, and there are still at least three aspects that warrant further dissections: (i) the development of stimuli for the treatment of immune paralysis in cancer or sepsis among infants and the elderly, and the potential of stimuli in protecting against COVID-19 and prevention of surgical site infections; (ii) the regulation of deleterious consequences of maladaptive programs like autoinflammatory and autoimmune diseases; (iii) more researches into nonspecific effects of vaccination program in the presence of pre-existing immunity to exert an impact on overall mortality [16, 139, 146, 147]. Only through continuous efforts to accomplish these investigations can we have a more comprehensive understanding of the trained immunity to utilize its potential better to serve our health.

## Data Availability

Not applicable.

## Abbreviations

ATF3: Activating transcription factor 3
BCG: Bacillus Calmette–Guérin
2-DG: 2-Deoxyglucose
DAMPs: Damage associated molecular patterns
DC: Dendritic cells
DMR: Differentially methylated region
GM-CSF: Granulocyte–macrophage; colony-stimulating factor
H3K4me3: Trimethylation of lysine 4 at histone 3
H3K4me1: Mono-methylation of histone 3 at lysine
H3K27ac: Acetylation of lysine 27 at histone 3
HIF1α: Hypoxia-inducible factor 1α
HMGB1: High-mobility group protein B1
HPV: Human papillomavirus
HSPs: Heat shock proteins
HSPCs: Hematopoietic stem and progenitor cells
HSV2: Herpes simplex virus type 2
IL: Interleukin
IFNs: Interferons
IRG1: Immune-responsive gene 1
IκB: Inhibitor of NF-κB
JMJD3: Jumonji domain-containing protein 3
KDM5: Histone lysine-specific demethylase 5
MDP: Muramyl dipeptide
MDSCs: Marrow-derived suppressor cells
mTOR: Mechanistic target of rapamycin
NFAT: Nuclear factor of activated T cells
NF-κB: Nuclear factor kappa-B
NK: Natural killer
NOD2: Nucleotide-binding oligomerization domain-containing protein 2
NRF2: Nuclear factor E2-related factor 2
PAMPs: Pathogen associated molecular patterns
ROS: Reactive oxygen species
RSV: Respiratory syncytial virus
SCID: Severe combined immunodeficiency
Set7: Lysine methyltransferase
TAMs: Tumor-associated macrophages
TCA: Tricarboxylic acid cycle
TME: Tumor microenvironment
TNF: Tumor necrosis factors
DR5–MLL1: WD repeat-containing protein 5–mixed lineage leukemia protein 1
